# Supplementary material for: Genetic Deficiency of the Histamine H4-Receptor Reduces Experimental Colorectal Carcinogenesis in Mice
Source: Cancers (Basel). 2020 Apr 8;12(4):912. doi: 10.3390/cancers12040912 (PMC7226035; doi:10.3390/cancers12040912)
Supplement: Supplementary file 1 [file cancers-12-00912-s001.zip › cancers-743506-supplementary.docx]

Article

Genetic Deficiency of the Histamine H_4_-Receptor Reduces Experimental Colorectal Carcinogenesis in Mice

Bastian Schirmer, Tamina Rother, Inga Bruesch, Andre Bleich, Christopher Werlein, Danny Jonigk, Roland Seifert and Detlef Neumann


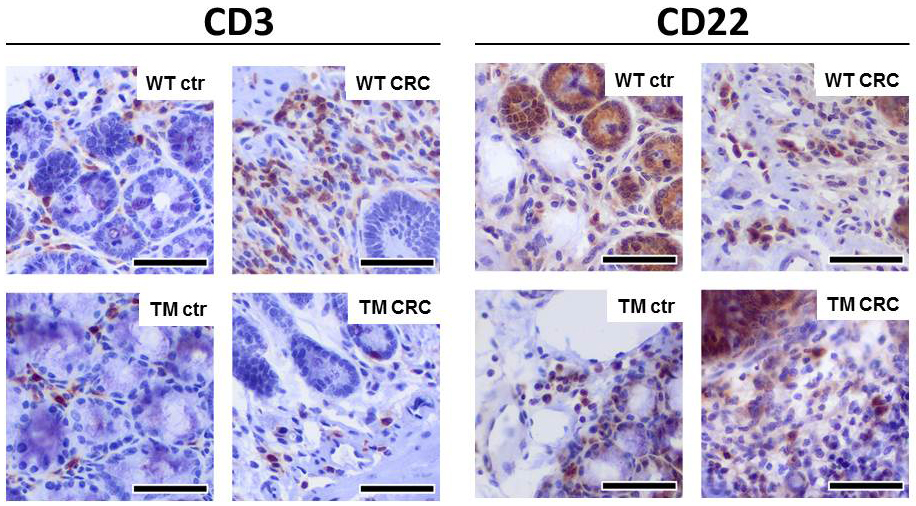


**Figure S1**. Colon tissues prepared out of the mice described in Figure 1 were fixed in buffered formalin, embedded in paraffin, and sliced into histological sections. Sections were stained for either CD 3 (Dako, Code A 0452, [Copenhagen](javascript:;), [Denmark](javascript:;)) or CD 22 (Zytomed Systems Cat.-No 503, [Berlin](javascript:;), [Germany](javascript:;)) and counterstained with haemalaun. Shown are representative photographs of the immunohistochemical analyses. WT: wild type, TM: H_4_R^−/−^, ctr: AOM-treated mice, CRC: AOM/DSS-treated mice.

| 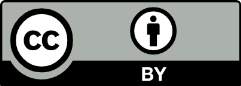 | © 2020 by the authors. Licensee MDPI, Basel, Switzerland. This article is an open access article distributed under the terms and conditions of the Creative Commons Attribution (CC BY) license (http://creativecommons.org/licenses/by/4.0/). |
| --- | --- |
